# Influence of expanded graphene on physical and chemical properties, and *in vitro* toxicity of glass ionomer cements for luting

**DOI:** 10.2340/biid.v13.45910

**Published:** 2026-05-26

**Authors:** Sarah Pereira Martins, Carolina Mara Geraldino Monteiro, Renan Rocha da Silva, Andrea Vaz Braga Pintor, Marcela Baraúna Magno, Maria Augusta Visconti, Maria Teresa Villela Romanos, Livia Rodrigues de Menezes, Lucianne Cople Maia, Matheus Melo Pithon

**Affiliations:** a Federal University of Maranhão, São Luís, Maranhão, Brazil; b São Leopoldo Mandic College, Campinas, São Paulo, Brazil; c Federal University of Rio de Janeiro, Rio de Janeiro, Brazil; d Department of Pediatric Dentistry, Veiga de Almeida University, Rio de Janeiro, Brazil; e Department of Prosthetic Dentistry, Federal University of Rio de Janeiro, Rio de Janeiro, Brazil; f University of Campinas (UNICAMP), Campinas, São Paulo, Brazil; g Department of Radiology, Federal University of Rio de Janeiro, Rio de Janeiro, Brazil; h Department of Microbiology and Immunology, Federal University of Rio de Janeiro, Rio de Janeiro, Brazil; i Institute of Macromolecules, Federal University of Rio de Janeiro, Rio de Janeiro, Brazil; j Department of Pediatric Dentistry and Orthodontics, Federal University of Rio de Janeiro, Rio de Janeiro, Brazil

**Keywords:** Glass ionomer cement, nanomaterials, chemical properties, expanded graphene

## Abstract

**Objective:**

To evaluate physical and chemical properties and *in vitro* toxicity of six luting glass ionomer cements (GICs) – Vidrion, Vitro Cem, Meron, Gold Label (GCGL), GC Fuji Plus (GCFP), and Riva Luting – incorporated with expanded graphene to correct the GICs’ deficiencies.

**Methodology:**

Expanded graphene was incorporated into GICs at concentrations of 0.25%, 0.5%, and 1%. Disc-shaped specimens (4 × 2 mm) were fabricated and submitted to an erosive challenge (EC) by immersion in 30 mL of lactic acid/lactate solution (pH 2.74) for 24 h at 37°C. Thickness, surface roughness (Ra), and microhardness were evaluated before and after EC. Fluoride release was measured in the acid solution. Radiopacity was evaluated in specimens (15 ± 1 mm; *n* = 3), and gray values were analyzed with ImageJ. Cytotoxicity was assessed by the neutral red uptake assay using L929 fibroblasts exposed to DMEM extracts.

**Results:**

No microhardness loss after EC was observed in Vitro Cem 0.25% (*p* = 0.056) and 0.5% (*p* = 0.457) or Meron 0.5% (*p* = 0.167), while Vidrion 0.5% showed increased hardness (*p* < 0.001). Surface roughness remained unchanged in Meron, GCGL, and Riva Luting. Riva Luting containing 0.25% (*p* < 0.01) and 1% rGO (*p* < 0.01) released more fluoride compared with the control. GCGL showed improved cell viability at all expanded graphene concentrations. Vidrion demonstrated greater radiopacity with expanded graphene addition, and Meron exhibited enhanced radiopacity at all concentrations. Worsening effects were observed, including reduced fluoride release in GCFP 1% (*p* < 0.05), decreased cell viability in GCFP (45% reduction), Meron, and Vitro Cem at all percentages, and increased microhardness loss in Vitro Cem 1%, Meron 0.5%, and GCFP at all concentrations.

**Conclusion:**

Expanded graphene incorporation produced positive, neutral, and negative effects depending on the GIC and concentration.

**KEY MESSAGES**

- The incorporation of expanded graphene into glass ionomer luting cements influenced their physicochemical and biological properties, with effects depending on the material and graphene concentration.
- Expanded graphene improved specific properties of certain materials, including increased microhardness in Vidrion, enhanced fluoride release in Riva Luting, improved radiopacity in Vidrion, and higher cell viability in GC Gold Label.
- Despite these improvements, some materials showed reduced fluoride release, decreased cell viability, or increased microhardness loss, indicating that the interaction between graphene and glass ionomer cement is material dependent.

## Introduction

In dentistry, luting cements are materials designed to perform multiple clinical functions and are widely used for the cementation of crowns, inlays, onlays, veneers, single and multiple-unit fixed prostheses, endodontic posts, and orthodontic bands in both orthodontics and pediatric dentistry. Understanding the properties and clinical indications of luting materials is essential to ensure the quality and longevity of cementation procedures.

In orthodontic band cementation, the primary role of the luting material is to seal the interface between the tooth surface and the band, providing adequate physical and chemical bonding. An effective seal is essential not only to maintain band retention but also to prevent microleakage at the tooth–band interface.

Glass ionomer cement (GIC) has been widely used for orthodontic band cementation due to its favorable properties, including a coefficient of thermal expansion compatible with dental tissues [1], chemical adhesion to enamel and dentin [2], sustained fluoride release [3], and biocompatibility [4]. However, conventional GICs present limitations such as low mechanical strength, inherent porosity, and limited esthetic performance when compared with resin-based materials [5], which has driven continuous efforts to enhance their physicochemical properties.

Several strategies have been proposed to improve GICs for luting (GIC-C) [6], including the incorporation of chitosan [7], ceramic additives [8], gum Arabic [9], and other modifying agents [10]. Despite these advances, there remains a demand for a luting material that is biocompatible and non-cytotoxic, radiopaque, does not adversely affect fluoride release, does not increase surface roughness, and exhibits adequate working and setting times. To date, no luting material fulfills all the characteristics of an ideal cement.

Graphene has emerged as a promising material in this context. Since its isolation by Novoselov et al. in 2004 [11], graphene has attracted significant attention due to its exceptional mechanical, thermal, electrical, and antibacterial properties. The incorporation of graphene into GICs for cementation is a relatively recent approach and has shown promising improvements in mechanical, tribological, and antibacterial performance [12–14].

Among the various graphene derivatives, expanded graphene was selected in the present study due to its derivation from graphite and the cost-effectiveness of the reduction method employed. Moreover, Brazil’s abundant graphite reserves – representing a substantial proportion of the global supply – highlight the strategic relevance of advancing graphene-based technologies. This study hypothesizes that the incorporation of expanded graphene does not adversely affect the physicochemical properties of the glass ionomer luting cements and may improve their inherent limitations. Therefore, the aim of this study was to evaluate the behavior of rGO-modified glass ionomer luting cements with respect to acidic erosion, fluoride release, radiopacity, and cytotoxicity.

## Methodology

### Study design

The experimental procedures in this *in vitro* study were conducted by previously trained operators. The six luting GICs tested are described in Table 1. Each material group was subdivided into a control group (0% – without incorporation of rGO) and the three different incorporations 0.25%, 0.5% and 1.0%. The sample sizes adopted for the erosive challenge (EC) test (*n* = 6) and for the radiopacity evaluation (*n* = 3) were based on the ISO 9917-1:2018 recommendations. Thickness, microhardness, and linear surface roughness (Ra) were measured before and after the EC, while fluoride release was assessed by quantifying the amount of fluoride released into the solution after the 24-hour immersion period.

**Table 1:** Description of glass ionomer cements used in the study.

Material	Manufacturer	Batch	Composition	Manipulation time	Curing time
Meron	VOCO, Cuxhaven, Germany	2052268	Powder: Aluminum fluorosilicate glass, polyacrylic acid and pigments Liquid: 10% tartaric acid solution	3 min	3–5 min
GC Gold Label	GC Corporation, Bunkyo-ku, Japan	2107081	Powder: alumino-silicate glass, polyacrylic acid powder Liquid: distilled water, polyacrylic acid, poly-basic carboxylic acid	4 min	3–4 min
Riva Luting	SDI, Melbourne, Australia	11616193	Powder: Fluoroaluminum silicate glass and polyalkenoic acid Liquid: Polyacrylic acid, tartaric acid, oxalic oxide and water	2 min	4 min
GC Fuji Plus	GC Corporation, Bunkyo-ku, Japan	2110011	Powder: alumino silicate glass Liquid: polyacrylic acid, distilled water, 2-hydroxyethyl methacrylate (HEMA), dimethacrylate	2 min 30 s	4 min 30 s
Vitro Cem	DFL, Rio de Janeiro, Brazil	21060695	Powder: Strontium Fluoride Aluminum Silicate, Dehydrated Polyacrylic Acid and Iron Oxide Liquid: Polyacrylic Acid, Tartaric Acid and Distilled Water	2 min 30 s	3–4 min
Vidrion C	DFL, Rio de Janeiro, Brazil	100321	Powder: Sodium Calcium Aluminum Fluorosilicate, Polyacrylic Acid Liquid: Tartaric Acid, Distilled Water	3 min	8 min

### Preparation of expanded graphene

Oxidized graphene was obtained by adding 0.5 g of natural graphite to the Erlenmeyer containing a mixture of concentrated sulfuric acid and nitric acid, in a ratio of 4:1 v/v, respectively, under reflux at a temperature of 80°C. The system was kept in agitation for 1 h. At the end, the oxidized graphite was centrifuged at 4,000 rpm for 10 min for decantation and washed with deionized water. After washing, the graphite was dried in an oven at 120°C for 1 h, and then the graphite was expanded in a muffle furnace at 1,000°C for 1 min. The expanded graphene powder obtained was incorporated into the GIC.

### Incorporation of graphene into GIC-C

Expanded graphene and GIC-C were weighed on analytical scales, and proportions of 0.25%, 0.5%, and 1% of OGr were incorporated into each of the six materials tested. The mixtures were stored in Falcon tubes, identified by an operator, and taken to the homogenizer for 15 min at a speed of 22 rpm (Phoenix Luferco, Model AP 22/28/32).

### Assessment of initial thickness, microhardness, and linear surface roughness

Six specimens (4 mm × 2 mm) from each group were made in silicone molds and stored in the refrigerator for 24 h in an Eppendorf with a damp cotton ball for complete attachment of the material. Samples with air failures or inclusions were rejected. Five specimen mold points were measured with a micrometer (Syntek, model 25ABS, digital) to calculate the initial thickness. After the measurements, the specimens were fixed in 30 × 30 mm blocks using double-sided tape. Initial Vickers microhardness (100 gf load applied for 15 s) was then measured using a microhardness tester (Micromet 2003, model no. 16005300; Buehler Ltd., Lake Bluff, IL, USA), followed by the assessment of initial linear surface roughness (Ra) using an optical profilometer (Nanovea PS50 Optical, NANOVEA^®^, Irvine, CA, USA). The specimens were subsequently returned to Eppendorf tubes for storage.

### Erosive challenge test

After 24 h, each specimen was immersed horizontally in an individual container with 30 ml of the erosion solution (lactic acid and lactate). The erosive solution was made with lactic acid and lactate, both previously calculated and dissolved in water. The pH of the solution was 2.74. It was then stored for 24 h in an incubator at (37°C ± 1°C). After 24 h of immersion, the specimens were removed, washed with water (as defined in ISO 3696:1987), and again subjected to five thickness measurements, Ra (Nanovea PS50 Optical, NANOVEA^®^, Irvine, USA) and microhardness (Micromet 2003, model no. 16005300; Buehler Ltd., Lake Bluff, IL, USA) assessments. The acid was stored for fluoride release reading in a container.

### Fluoride release

For fluoride dosage (*n* = 6), first, the calibration curve was obtained using the blank, standard solutions, and the test solution. Blank was first used to zero the equipment (Thermo Fisher Scientific, Waltham, MA, USA). Next, the solutions at 0.25, 0.5, 1.0, 2.0, 4.0, 8.0, and 16.0 (TISAB and fluoride solution) were read, and finally, the test solution (known solution – 1 ppm fluoride – Thermo Fisher Scientific, Waltham, MA, USA) was read. The entire procedure described was performed three times. After this stage was completed, the samples were read. For the reading, 100 uL of sample + 900 uL of water + 1,000 uL of TISAB (buffer solution) were used. The reading was carried out in duplicate.

### Cytotoxicity

For the cytotoxicity assay, L929 cell culture, mouse fibroblast cells, obtained from the American Type Culture Collection (ATCC, Rockville, MD) (mouse fibroblast) was used. The cells were maintained in Dulbecco-modified Eagle Medium (DMEM) (Vitrocell^®^, Campinas, Brazil) plus 0.03% glutamine – 2 mM (Sigma-Aldrich^®^, St. Louis, USA), 200 U/mL penicillin and streptomycin (HyClone Laboratories, USA), 2.5 mg/mL amphotericin B (HyClone Laboratories, USA), 0.25% sodium bicarbonate solution (Sigma-Aldrich^®^, St. Louis, USA), 4-(2-hydroxyethyl)-1-piperazineethanesulfonic acid – HEPES (Alamar Tecno Científica^®^, São Paulo, Brazil) 10 mM, and 10% fetal bovine serum (FBS) (Cultilab – Campinas – Brazil), and incubated at 37°C, in an environment containing 5% CO_2_.

One specimen per group (*n* = 1) was prepared in the form of a 4 mm × 2 mm disc. The specimen was immersed in 1 mL of DMEM in a 24-well plate for 7 days at a temperature of 24–26°C to obtain the extract. Subsequently, 100 μL of the supernatant was collected and added to a monolayer of L929 cells cultured in 96-well plates. The cells were incubated for 4 h at 37°C, after which the supernatant was replaced with DMEM. Cell viability was then evaluated after 24 h of incubation using the neutral red ‘dye-uptake’ technique [15], with minor modifications.

After this period, 100 μL/well of 0.01% neutral red solution (in maintenance medium) was added, and the cells were incubated for 2 h at 37°C in an atmosphere containing 5% CO₂. The supernatant was subsequently discarded, and the monolayer was washed with 200 μL/well of phosphate-buffered saline (PBS, pH 7.2). Then, 100 μL/well of a solution containing 1% acetic acid (Vetec, Rio de Janeiro, Brazil) and 50% methanol (Reagen, Rio de Janeiro, Brazil) was added to disrupt the cells and release the incorporated dye. Absorbance was measured using a spectrophotometer (BioTek, Winooski, VT, USA) at a wavelength of 492 nm. The extract obtained from the specimen was tested in technical triplicates, and the percentage of viable cells was calculated from the mean optical density (OD) of the three readings relative to the mean OD of the control cells, which was considered 100% cell viability.

### Radiopacity

#### Preparation of samples

The mold (15 mm × 1 mm) was positioned and filled until excess cement was obtained. A sheet of plastic film was placed over the material in the mold and covered with a glass plate (10 mm), thus making the excess material come out. This assembly was placed inside the oven at 37°C for 30 min. The sample was removed and inserted into a plastic container containing 5 ml of reverse osmosis for 24 h in an oven at a temperature of 23 ± 1°C. The samples were then measured in their center with a micrometer to verify whether they were within the correct thickness range (1.0 ± 0.1 mm). Samples that were above the measurement were worn with #400 sandpaper until they were within the specified range. This test was carried out following the recommendations of ISO 9917-1:2018 (*n* = 3).

#### Radiographic acquisition and image analysis

The specimens (*n* = 3) were radiographed using the Express digital imaging system (Kavo; Instrumentarium) with the Focus Dental X-ray machine (Instrumentarium Dental, Tuusula, Finland) operating at 70 kVp, 7 mA, 40 cm source- object distance, and with an exposure time of 0.03 s. To ensure the reproducibility of the gray values, each specimen was radiographed separately. All radiographic images were exported in TIFF format with 8-bit contrast resolution. Gray values were obtained from each material using ImageJ, a public domain software developed by the National Institutes of Health (NIH-USA). A quadrangular region of interest (ROI), of dimensions 0.8 × 0.8 mm, was established for each step of the scale to be then plotted against the corresponding thickness in millimeters using the Microsoft Excel scatter plot. The trend equation between these values was established for each radiograph (*n* = 72), corresponding to each material (e.g. y = 8.9826x + 132.26 and R2 = 0.98; where y = gray value e.g. = mm of aluminum).

### Statistical analysis

Statistical analysis was initially performed using descriptive statistics for both control and experimental groups. Data normality was assessed using the Shapiro–Wilk test, with a significance level set at *p* < 0.05. Comparisons between measurements obtained before and after the EC were conducted using the paired Student’s *t*-test for normally distributed data and the Wilcoxon signed-rank test for non-normally distributed data. In addition, the association between microhardness and thickness was evaluated using Spearman’s rank correlation analysis. For all statistical tests, the significance level was set at 5%.

## Results

### Erosive challenge – microhardness, thickness, and Ra

#### Microhardness

Microhardness results before and after the EC are shown in Table 2. Most GICs, with and without graphene, showed a reduction in microhardness after EC (*p* < 0.05). Only Vidrion with 0.5% graphene showed an increase in the mean microhardness (*p* < 0.001). Vitro Cem (control, 0.25% and 0.5% graphene), Riva (control), and Meron (0.5% graphene) showed no difference in their microhardness after EC (*p* > 0.05).

**Table 2:** Mean microhardness ± standard deviation of the different glass ionomer cements (GICs), without and with different percentages of graphene incorporation.

GIC-C	0%	0.25%	0.5%	1%
**GC GL**				
Pre Er	39 ± 6.25	36 ± 5.98	35.7 ± 4.72	35.1 ± 8.67
Post Er	16.8 ± 5.42	16.8 ± 2.68	18.4 ± 2.16	15.7 ± 2.37
*p*-value	< 0.001 W	< 0.001 t	< 0.001 W	< 0.001 t
**Vidrion**				
Pre Er	23.1 ± 9.26	26.4 ± 4.5	**10.4 ± 2.77**	26.2 ± 4.53
Post Er	15.3 ± 2.67	16.4 ± 4.77	**17.3 ± 2.47**	14.2 ± 3.11
*p*-value	< 0.001 W	< 0.001 t	< 0.001 W*	< 0.001 W
**Vitro Cem**				
Pre Er	16.6 ± 2.89	19.8 ± 2.79	18.5 ± 2.78	23 ± 6.47
Post Er	16 ± 2.6	17.9 ± 3.27	18.9 ± 2.73	17.2 ± 1.35
*p*-value	0.392 t	0.056 t	0.457 t	0.002 t
**Riva**				
Pre Er	28.9 ± 2.93	25.6 ± 2.54	22.9 ± 2.78	25.3 ± 2.41
Post Er	29.9 ± 12.7	19.7 ± 2.73	18.6 ± 2.74	20.3 ± 5.06
*p*-value	0.899 W	< 0.001 t	< 0.001 t	0.007 W
**Meron**				
Pre Er	26.8 ± 5.23	17.5 ± 4.17	19.7 ± 3.54	18.1 ± 5.62
Post Er	17.7 ± 4.2	12.6 ± 4.27	18 ± 3.78	9.71 ± 2.49
*p*-value	< 0.001 W	0.002 W	0.167 W	< 0.001 W
**GC Fuji Plus**				
Pre Er	29.1 ± 2.51	20.1 ± 3.34	22.3 ± 2.59	18.6 ± 2.85
Post Er	15.9 ± 1.64	15.7 ± 1.57	16.2 ± 2.54	14.9 ± 1.27
*p*-value	< 0.001 t	0.001 W	< 0.001 t	< 0.001

Pre Er = before erosive challenge; Post Er = after erosive challenge. *W* = Wilcoxon test, *t* = paired *t*-test.

Note: Asterisks (*) mean statistical significance followed by microhardness gain (*p* < 0.05).

**Figure 1:**
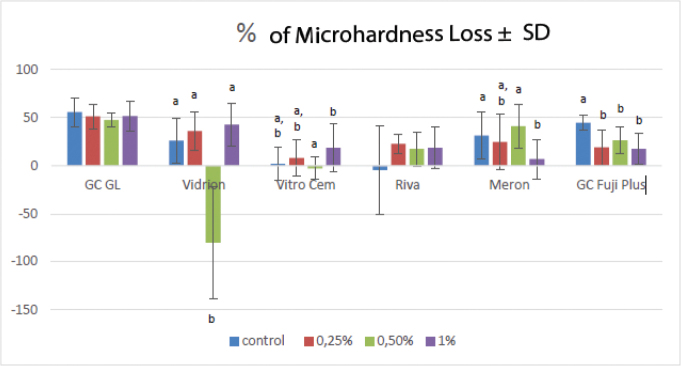
Mean percentage of MH loss ± standard deviation of the different GICs. The letters *a* and *b* refer to the intragroup comparison.

Figure 1 shows the values of microhardness loss in each group. Compared to their controls, the incorporation of graphene did not influence the percentage of microhardness loss for the GCGL and Riva Luting (*p* > 0.05). However, the Vitro Cem 1%, Meron 0.5%, and GC Fuji Plus 0.25%, 0.5%, and 1% graphene showed a higher percentage of microhardness loss (*p* < 0.05). The incorporation of 0.5% graphene in Vidrion resulted in microhardness gain (*p* < 0.001).

#### Thickness

The results regarding the thickness of each group, before and after EC, are presented in Table 3. It can be observed that GCGL, Vidrion, Vitro Cem, and Riva, with and without graphene, showed a reduction in their thickness after EC (*p* < 0.05). On the other hand, the Meron groups containing graphene showed increased thickness after the EC at 0.25% (*p* = 0.018), 0.5% (*p* = 0.008), and 1% (*p* = 0.026). Similarly, Fuji Plus showed increased thickness in the control group and in the graphene-containing groups – 0.25% (*p* = 0.008), 0.5% (*p* < 0.001), and 1% (*p* < 0.001). Only the control Meron did not show a difference in its thickness after EC (*p* > 0.05).

**Table 3:** Mean thickness ± standard deviation of the different glass ionomer cements (GICs), with and without different percentages of expanded graphene incorporation.

GIC	0%	0.25%	0.5%	1%
**GC GL**				
Pre Er	1.96 ± 0.08	1.96 ± 0.09	2.0 ± 0.07	2.0 ± 0.03
Post Er	1.88 ± 0.06	1.88 ± 0.05	1.94 ± 0.05	1.91 ± 0.06
*p*-value	0.014 t*	0.017 t*	0.031 t*	0.013 t*
**Vidrion**				
Pre Er	2.09 ± 0.08	2.17 ± 0.03	2.03 ± 0.05	2.13 ± 0.04
Post Er	1.85 ± 0.07	1.87 ± 0.06	1.86 ± 0.05	1.84 ± 0.06
*p*-value	< 0.001 t*	< 0.001 t*	< 0.001 t*	< 0.001 t*
**Vitro Cem**				
Pre Er	1.96 ± 0.03	1.88 ± 0.05	1.91 ± 0.08	1.90 ± 0.07
Post Er	1.63 ± 0.07	1.56 ± 0.08	1.59 ± 0.08	1.54 ± 0.06
*p*-value	< 0.001 t*	< 0.001 t*	< 0.001 t*	< 0.001 t*
**Riva**				
Pre Er	2.02 ± 0.08	2.02 ± 0.07	2.3 ± 0.03	1.99 ± 0.06
Post Er	1.83 ± 0.07	1.85 ± 0.06	1.88 ± 0.03	1.83 ± 0.06
*p*-value	< 0.001 t*	< 0.001 t*	< 0.001 t*	< 0.001 t*
**Meron**				
Pre Er	1.95 ± 0.08	1.89 ± 0.07	1.92 ± 0.05	1.93 ± 0.08
Post Er	1.96 ± 0.05	1.94 ± 0.09	1.99 ± 0.04	1.98 ± 0.06
*p*-value	0.708 t	0.016 t*	0.003 t*	0.025 t*
**GC Fuji Plus**				
Pre Er	2.0 ± 0.06	1.95 ± 0.06	1.93 ± 0.06	1.93 ± 0.07
Post Er	2.12 ± 0.08	2.1 ± 0.06	2.08 ± 0.08	2.06 ± 0.06
*p*-value	0.003*	0.006*	< 0.001*	< 0.001*

Pre Er = before erosive challenge; Post Er = after erosive challenge; *t* = paired *t*-test.

Note: The asterisks (*) indicate an increase or decrease in thickness with statistical significance (*p* < 0.05).

**Figure 2:**
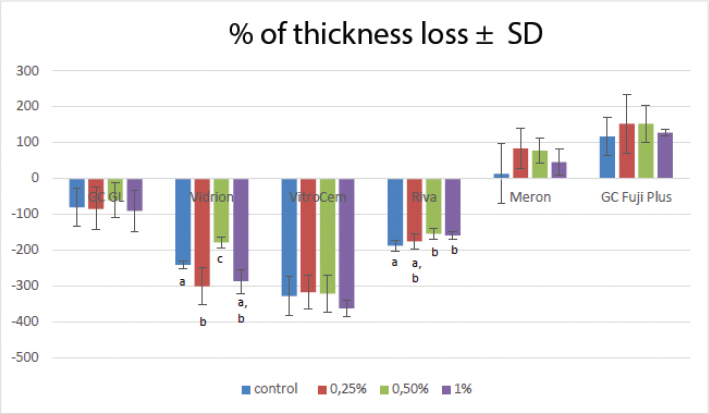
Mean percentage of thickness loss ± standard deviation (SD) of the different GICs. The letters *a*, *b*, and *c* refer to the intragroup comparison.

Figure 2 shows the values of % thickness loss in each group. Compared to their controls, the 0.5% Vidrion and the 0.5% and 1% Riva showed a reduction in their thickness (*p* < 0.05). Regarding the intergroup comparisons, a statistically significant difference was observed between the control GICs and all graphene incorporations (*p* < 0.001). Vitro Cem showed the highest percentage of thickness loss in both the control group and the groups containing 0.25%, 0.5%, and 1% graphene.

#### Correlation between thickness and hardness

**Figure 3:**
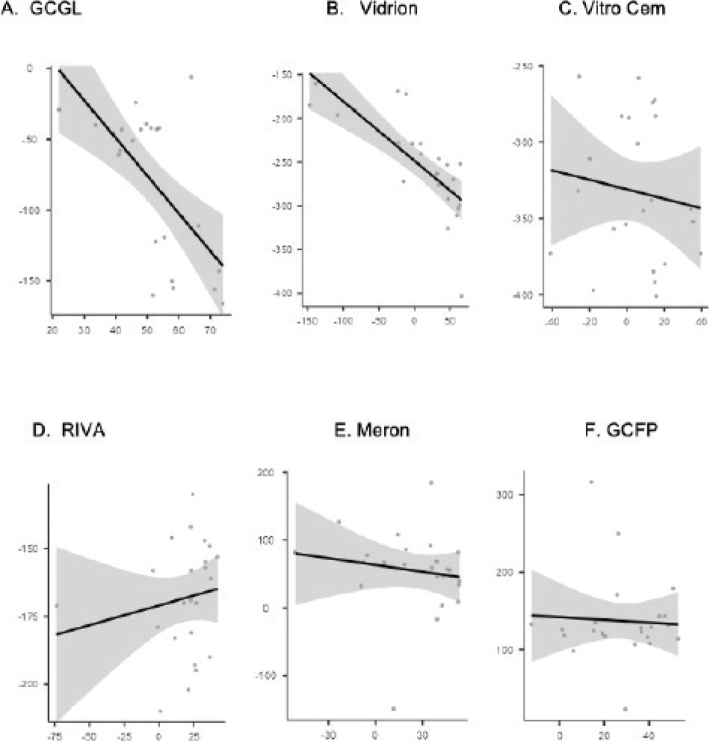
Correlation between the percentage of thickness loss and the microhardness of each glass ionomer cement. The correlation was evaluated using Spearman’s correlation test.

Vidrion was the only CIV-C that showed a strong and negative correlation between % of microhardness loss and thickness (*r* = −0.844; *p* < 0.001) after EC (Figure 3).

#### Profilometry – linear surface roughness analysis (Ra)

Regarding roughness after EC, in the intragroup comparison, there was no statistical difference in Meron (*p* > 0.05), GCGL (*p* > 0.05), and Riva Luting (*p* > 0.05) groups (Table 4). There was a worsening of the surface roughness of Vitro Cem 0.5% (*p* < 0.001), Vidrion 1% (*p* < 0.001), and GCFP 0.25% (*p* = 0.010) and 0.5% (*p* = 0.026) compared to their controls. The Ra value (Ra, μm) was used because it represents the arithmetic mean value of all absolute distances from the centerline roughness profile within the measurement length.

**Table 4:** Mean and standard deviation (SD) of the Linear Roughness Profile (Ra) of each control and graphene-incorporated glass ionomer cements (GICs).

GIC	0%	0.25%	0.5%	1%
**Vitro Cem**				
Ra Pre	0.361 ± 0.06	1.62 ± 5.04	1.34 ± 3.85	0.509 ± 0.137
Ra Post	2.10 ± 2.84	23.1 ± 29.3	63.7 ± 49.6	27.0 ± 34.9
*p*-value	< 0.001*	0.257	< 0.001*	0.196
**Meron**				
Ra Pre	3.41 ± 4.75	0.637 ± 0.965	0.555 ± 0.123	0.637 ± 0.965
Ra Post	49.9 ± 96.5	37.5 ± 27.4	29.7 ± 35.4	37.5 ± 31.7
*p*-value	< 0.001*	0.656	0.585	0.103
**Vidrion**				
Ra Pre	0.807 ± 0.128	1.28 ± 1.49	0.820 ± 0.08	1.04 ± 1.24
Ra Post	30.0 ± 39.8	16.6 ± 21.4	6.29 ± 7.70	2.28 ± 3.15
*p*-value	< 0.001*	0.426	0.055	**< 0.001**
**GC GL**				
Ra Pre	0.803 ± 2.01	0.689 ± 1.42	0.288 ± 0.05	0.372 ± 0.05
Ra Post	3.79 ± 5.13	2.77 ± 4.72	13.1 ± 27.8	3.93 ± 5.27
*p*-value	< 0.001*	0.761	1.000	0.987
**Riva**				
Ra Pre	0.305 ± 0.05	0.433 ± 0.08	1.63 ± 3.84	0.284 ± 0.03
Ra Post	30.5 ± 110	5.44 ± 5.85	15.6 ± 24.5	2.43 ± 3.62
*p*-value	< 0.001*	0.992	0.999	0.110
**GC Fuji Plus**				
Ra Pre	0.304 ± 0.03	0.353 ± 0.04	0.463 ± 1.39	0.773 ± 1.9
Ra Post	2.87 ± 3.51	2.92 ± 2.69	1.67 ± 3.2	1.38 ± 2.19
*p*-value	< 0.001*	1.000	0.010*	0.026*

The comparison of incorporated groups was made based on the control.

Note: Asterisks (*) mean statistical significance (*p* < 0.05).

### Fluoride release

Only GCFP and Riva had all groups with a normal distribution. There was no statistically significant difference in the Meron, Vidrion, and GC Gold Label groups (*p* > 0.05) (Table 5). In the GC Fuji Plus group, there was a reduction in fluoride release between the control and the 1% group (*p* < 0.05), and in Riva Luting there was an increase in fluoride release in the 0.25% (*p* < 0.01) and 1% (*p* < 0.001) groups, as well as in the Vitro Cem 0.5% group (*p* < 0.05).

**Table 5:** Mean and standard deviation (SD) of fluoride release for each glass ionomer cement (GIC).

GIC	0%	0.25%	0.5%	1%
**Vitro Cem**	55.0 ± 3.64	50.9 ± 2.21	54.1 ± 6.37	47.4 ± 3.42
*p*-value	–	0.410	*p* < 0.05*	0.878
**Meron**	16.8 ± 2.48	18.5 ± 3.00	18.1 ± 4.21	16.7 ± 1.13
*p*-value	–	0.677	0.999	0.989
**Vidrion**	40.0 ± 13.4	32.0 ± 14.6	23.7 ± 5.59	21.8 ± 4.03
*p*-value	–	0.772	0.219	0.112
**GC GL**	16.6 ± 7.98	25.7 ± 12.2	19.8 ± 4.66	21.1 ± 13.7
*p*-value	–	0.292	0.575	0.989
**Riva**	23.7 ± 1.08	24.9 ± 0.774	32.6 ± 3.19	32.7 ± 1.60
*p*-value	–	0.122	*p* < 0.01*	*p* < 0.001*
**GC Fuji Plus**	33.0 ± 1.47	38.2 ± 4.96	32.6 ± 2.62	30.4 ± 0.545
*p*-value	–	0.512	0.438	*p* < 0.05*

The *p*-value is compared to the material with 0% expanded graphene (control).

Note: Asterisks (*) mean statistical significance (*p* < 0.05).

### Cytotoxicity

The reading of the cell viability of the Vitro Cem 0.5% and Vidrion 0.5% and 1% groups was not possible due to fungal contamination. It was observed that the GCGL group improved cell viability to 100% at all concentrations (0.25%, 0.5% and 1%). However, there was a reduction in the cell viability in the GCFP, Meron, and Vitro Cem groups in all graphene incorporations, and in the Riva Luting group, only the 0.5% incorporation remained stable (Table 6).

**Table 6:** Triplicate analysis of the mean percentage of cell viability after 24 h using the neutral red uptake assay.

GIC	0%	0.25%	0.5%	1%
**Vitro Cem**	35	20*	20	*
**Meron**	57	26	45	40
**Vidrion**	32	34	*	*
**GC GL**	85	100	100	100
**Riva**	43	35	44	25
**GC Fuji Plus**	90	42	65	63

GIC: glass ionomer cement.

Note: Asterisk (*) means contamination.

### Radiopacity (gray values)

Descriptive analysis showed that the Vidrion group presented higher gray values as the graphene concentration increased. In the GCFP and GCGL groups, higher graphene concentrations resulted in lower gray values (less radiopaque). The Riva Luting and Vitro Cem groups showed lower gray values at the concentration of 0.25% (Table 7).

**Table 7:** Mean ± standard deviation (SD) of radiopacity measured in mmAl.

GIC	0%	0.25%	0.5%	1%
**Vitro Cem**	2.10 ± 0.40	1.23 ± 0.27*	2.03 ± 0.37	2.85 ± 0.07
**Meron**	–0.53 ± 0.57	–0.12 ± 0.83	0.38 ± 0.31	0.09 ± 1.00
**Vidrion**	1.00 ± 0.36	2.60 ± 0.24	2.65 ± 0.38	3.28 ± 0.39
**GC GL**	5.73 ± 0.66	4.22 ± 0.36	4.07 ± 0.42	3.60 ± 0.35
**Riva**	3.56 ± 0.35	1.70 ± 0.55*	3.51 ± 0.74	2.13 ± 0.26
**GC Fuji Plus**	4.96 ± 0.61	4.91 ± 0.19	4.23 ± 0.48	3.83 ± 0.45

GIC: glass ionomer cement.

Note: Asterisk (*) means reduction of radiopacity based on the mean ± standard deviation (SD).

## Discussion

GIC is widely used in dental practice; however, it still presents some limitations previously described in the introduction of this study. Therefore, the present investigation evaluated the effect of expanded graphene incorporation (0.25%, 0.5%, and 1%) into luting GICs under EC conditions. Overall, the results demonstrated that graphene incorporation influenced the physicochemical and biological behavior of the materials depending on the cement formulation and the graphene concentration used. Increased microhardness was observed in Vidrion containing 0.5% graphene, while fluoride release was enhanced in Riva Luting with 0.25% and 1% graphene. In addition, cell viability reached 100% in the GCGL group at all concentrations, and Vidrion showed increased radiopacity in the presence of graphene. However, some negative effects were also observed, including reduced fluoride release and decreased cell viability in specific materials. Therefore, the study hypothesis was partially accepted.

The EC in this study was performed using lactic acid to simulate gastric acid exposure, and it showed several results to be explored. In comparison with the erosive assay by Oliveira et al. [16], conducted with hybrid GIC and showing that neither microhardness nor surface roughness (Ra) was altered after EC with Coca-Cola, similar findings were observed in the present study. Specifically, similar results for microhardness were found in the Vitro Cem (0.25% and 0.5%) and Meron (0.5%) groups, while for surface roughness, similarities were observed in the GCFP, Vitro Cem, and Vidrion groups. In another erosive test [15], hybrid GIC immersed in HCl for 30 h exhibited inferior performance in surface roughness (Sa), roughness profile (Rv), surface loss (SL), and microhardness (MI) when compared to resin-based materials. Thus, under gastric acid exposure (HCl), this material may be clinically less favorable, and resin-based materials may be more appropriate for patients with gastroesophageal reflux disease. It is also worth mentioning the Vidrion 0.5% that increased the hardness after EC. This material was the only one that showed a high correlation between thickness loss and hardness gain. Therefore, it is suggested that SL of the GIC may have exposed graphene-rich subsurface regions, forming a potential ‘graphene barrier’. Due to the high mechanical strength and stiffness of graphene, this subsurface layer may have contributed to the increased microhardness values observed in this group. As for roughness, it is known that it is directly influenced by the structure of the matrix and by the characteristics of the particles of each material. Therefore, the composition of each material determines its behavior under acid challenge.

Solubility is an important factor for dental materials. GICs in the oral environment are in full contact with different fluids, including saliva, gingival crevicular fluid, and water. Through the functional activity of the oral cavity, the surface of the GICs can gradually dissolve and cause damage to the materials. GIC is sensitive to water in the critical first 24 h. High solubility and initial syneresis or imbibition can result in dimensional alteration, pore formation, and reduced mechanical properties [17, 18]. This dimensional change corroborates the results of this study, as they showed a change in thickness after erosion in all groups (including the control), with the exception of Meron without graphene (*p* > 0.05), demonstrating once again that GICs have high sensitivity to moisture, which makes them unstable and soluble in immersion challenges. The presence of graphene increased the thickness of Meron in all percentages.

Regarding fluoride release, older studies have suggested that the total amount of fluoride is less than 1% of the available fluoride content in cement [19]. Thus, fluoride release is not simply a function of fluoride content but is dependent on the composition of the cement and the kinetics of its setting reaction, crucial factors in determining the amount of fluoride released. According to the method used in this study, graphene did not alter the fluoride release pattern of the respective controls in any GIC; only in the Riva Luting group, fluoride increased its release at concentrations of 0.25% and 1%. It is known that fluoride-releasing materials are unable to interfere with the formation of biofilm on the dental surfaces adjacent to them or to inhibit the production of acid by dental biofilms. However, the fluoride released slows down the progression of caries lesions on the tooth surfaces adjacent to dental materials [20]. A study evaluating fluoride release in graphene-incorporated [21] GICs demonstrated that fluoride concentration decreased over time in materials containing graphene. Although pristine graphene was used, differing from the expanded graphene employed in the present study, these findings remain relevant to the fluoride-release behavior of GICs. Fluoride release was assessed at 7, 21, and 28 days, with the highest fluoride peak observed at 7 days, followed by a gradual decrease over time. While the referenced study used deionized water as the storage medium, the present investigation evaluated fluoride release under acidic conditions using lactic acid. Therefore, further investigations assessing long-term fluoride release under acidic pH conditions are recommended to better elucidate the sustained preventive potential of graphene-modified GICs.

According to the cytotoxicity assay, the present study showed that only the GC Gold Label (GCGL) group exhibited a significant improvement in cytocompatibility among all graphene incorporations into GIC. A similar study by Liu et al. [22] reported no cytotoxic effects of graphene incorporation into GIC; however, this discrepancy may be explained by differences in the methodologies employed. While Liu et al. found no cytotoxicity in the presence of fluorinated graphene using the MTT assay, the present study used expanded graphene and the neutral red uptake assay. These discrepancies may be partly attributed to the cytotoxicity assays employed, as MTT reflects mitochondrial metabolic activity rather than true cell viability, whereas neutral red selectively stains viable cells. In addition, fluorinated graphene is more hydrophobic and exhibits lower surface reactivity [23], whereas expanded graphene presents higher electrical conductivity and stronger cellular interactions [24] and has been reported to induce greater cellular stress compared with more functionalized graphene derivatives [25]. The cytotoxicity results obtained in this study may provide greater clinical relevance and importance for the scientific community.

In the evaluation of the radiopacity, only Vidrion showed increased radiopacity with increasing graphene concentration. The fact that some materials present lower gray values with graphene may not suggest a direct clinical implication when the material is used for orthodontic band cementation, and the main purpose of this test was the characterization of graphene incorporated into the luting GIC. Also, it is important to highlight that the specimen used had a thickness of 1 mm and clinically the thickness may be larger, leading to different gray values and radiopacity. In addition, the material exhibited darker gray values.

There were some limitations of this study. First, contamination occurred in the cytotoxicity test of Vitro Cem 0.5%, Vidrion 0.5% and 1% groups; and second, cytotoxicity and radiopacity were evaluated using descriptive analysis. Another point to consider is the negative effects observed after graphene incorporation, including reduced fluoride release in the GCFP 1% group, decreased cell viability in the GCFP, Meron, and Vitro Cem groups at all concentrations, and increased microhardness loss in the Vitro Cem 1%, Meron 0.5%, and GCFP groups. Therefore, further studies evaluating the chemical behavior of each GIC with graphene incorporation, considering its individual composition, are recommended. Clinically, the results of this study may affect treatment outcomes. Reduced microhardness and increased surface roughness may increase the risk of failure, require recementation, and affect treatment time. Fluoride release can influence the progression of caries lesions on dental surfaces. In addition, cytotoxicity evaluation is essential because the material must not be toxic to human cells [26].

Graphene presents promising future perspectives due to its intrinsic properties and potential to enhance dental restorative materials. When incorporated into GICs, graphene may improve physicochemical performance; however, comprehensive biological and physicochemical evaluations are required to clarify its clinical behavior. Future studies using ≥ 2% expanded graphene should evaluate both the physicochemical and biological behavior of these materials under long-term aging conditions. Such investigations should include cytotoxicity assays, microhardness, surface roughness, degradation under acidic and aging challenges, long-term fluoride release in saliva-simulating solutions, and radiopacity assessed using standardized protocols.

## Conclusion

This study demonstrated that the incorporation of expanded graphene into GIC increased the microhardness of Vidrion at 0.5%, enhanced fluoride release of Riva Luting at 0.25% and 1%, improved the cell viability of GCGL to 100% across all graphene-containing concentrations, and improved the radiopacity of Vidrion. While some parameters remained unchanged and others showed deterioration in the presence of graphene, most outcomes were either improved or unaffected. These findings indicate that graphene chemically interacts with the material, and this interaction warrants further investigation. The authors consider this study to have strong scientific rigor and to provide a reliable basis for future research exploring graphene as a strategy to overcome the limitations of GICs for luting.
